# Sources of Animal Heat

**Published:** 1891-10

**Authors:** A. D. Barr

**Affiliations:** Calamine, Ark.


### SOURCES OF ANIMAL HEAT.

---

BY A. D. BARR, M. D., CALAMINE, ARK.

---

The text-books, generally, treat animal heat as mainly due to chemical action, and that this action consists in the oxidation of the food consumed.

Also, that the amount of animal heat can be tolerably accurately calculated by ascertaining how much heat the food is capable of liberating when completely oxidized, but as the food is not completely oxidized in the body, the amount of heat that can be generated, which is contained in the excreta, must be deducted.

In an address delivered before the Ninth International Medical Congress, Prof. Austin Flint said:

"In a series of experiments made upon my own person, for twenty-four hours, under a liberal diet, I calculated the heat value of the food ingested as equal to 14, 979.15 heat units.

"At that time (1878), I weighed 186½ pounds, and according to my estimate I produced 17,880 heat units in twenty-four hours. There was no difference in the body weight at the beginning and at the end of the observation. These observations show that nearly one-sixth of the heat estimated as actually produced by the body, was not accounted for by the heat value of the food.

"There can be little question with regard to the accuracy of the accepted method for estimating the heat value of articles of food, and it follows logically that there must either be a grave error in the estimation of the heat produced by the body, or there are certain processes going on within the body not taken into account by physiologists, which involve a considerable production of animal heat. It is to be remembered, also, that I made no allowance for the conversion of a certain portion of the heat produced in the body into force expended in circulation, respiration, locomotion," etc.

As it is a well established fact that heat is generated in the body by the process of oxidation, and as the object of this article is to call attention to what I believe to be the sources of animal heat, I pass over that part which has been thoroughly established. As animal heat differs in no way from any other heat, and as any force that will produce heat without the body will also produce it within, it becomes necessary to examine the body and see if there are any forces within it that can be converted into heat, and if so it is to them we are to look for the explanation of the excess of heat produced within the body above that which is contained in the food, which was pointed out by Prof. Flint.

As the friction of the blood against the walls of the blood vessels and that of the muscles has been credited with the production of a certain portion of animal heat, I will pass over these forces also, without further notice.

### HEAT DUE TO PRESSURE.

If a body be compressed its temperature rises according to the diminution of its volume. In the body the entire force of the heart's action is converted into heat, and this force has been variously estimated at from 124 to 200 foot tons daily. In the case of the heart the force it contracts with drives the blood as far as the capillaries, by which time its force has been mostly expended in overcoming the pressure under which it labors, and when force is lost heat is generated; or rather the force is converted into heat. The effect of a division of the sympathetic nerve in the neck is to relax the arterioles of the side of the head corresponding to the lesion; those of the ear, for example, become more conspicuous and its temperature is considerably increased, and if the head is protected from external cold the venous blood, after division of the sympathetic, will be found to exceed in temperature that of the arterial.

Thus I believe, demonstrating the position I have taken, that the force of the heart's action is entirely converted into heat by the time the blood reaches the capillaries, and, is there partially reconverted into force, which serves to maintain the circulation. In the case of the division of the sympathetic nerve the arterioles become dilated as a self-evident consequence, as much force is not required to maintain the circulation, and the overplus of the force of the heart that was converted into heat is not reconverted into force, the consequence of which is, the venous blood returned from the region of the lesion is of higher temperature than that of the arterial blood which supplies it.

### HEAT DUE TO ABSORPTION.

Motacular action, for example absorption, is usually followed by a rise of temperature. Pouillet found when a liquid is poured on a pulverized solid heat is generated, and that the amount of heat thus produced varies according to the nature of the substance. With organic substances, as the metals, the increase is four-tenths of a degree, but with organic substances, as for example, starch or flour, the increase varies from one to ten degrees. The absorption of gases produces the same phenomenon. If the absorption of a fluid outside of the body is capable of generating heat, it should be conceded the same property within it, and if it be admitted that the absorption of a fluid is capable of liberating heat within the animal economy, and if the degree of heat so generated be comparable to that generated by the absorption of a fluid by such bodies as starch, flour, roots, dried membranes, etc., we have a source that must be credited with no inconsiderable place in the production of animal heat.

The constant absorption of gases that are constantly taking place in the animal body is of course likewise followed by the generation of heat. It is, I believe, only by the sources of heat here considered that the difference pointed out by Prof. Flint in the heat value of the food and the amount of heat actually generated by the animal body can be accounted for.

